# A 2D lightweight instrumented wheel for assessing wheelchair functionality/activity

**DOI:** 10.1177/20556683231155198

**Published:** 2023-02-07

**Authors:** Reto Togni, Manuel Müller, Stefan Plüss, William R Taylor, Roland Zemp

**Affiliations:** Laboratory for Movement Biomechanics, 27219 ETH Zürich, Zurich, Switzerland

**Keywords:** Manual wheelchair, instrumentation, force measurement, kinetics, human factors, activities of daily living, assessment

## Abstract

**Introduction:**

Force measurement wheels are essential instruments for analysing manual wheelchair propulsion. Existing solutions are heavy and bulky, influence propulsion biomechanics, and are limited to confined laboratory environments. In this paper, a novel design for a compact and lightweight measurement wheel is presented and statically validated.

**Methods:**

Four connectors between the push-rim and wheel-rim doubled as force sensors to allow the calculation of tangential and radial forces as well as the point of force application. For validation, increasing weights were hung on the push-rim at known positions. Resulting values were compared against pre-determined force components.

**Results:**

The implemented prototype weighed 2.1 kg and was able to transmit signals to a mobile recording device at 140 Hz. Errors in forces at locations of propulsive pushes were in the range up to ±3.1 N but higher at the frontal extreme. Tangential force components were most accurate.

**Conclusion:**

The principle of instrumenting the joints between push-rim and wheel-rim shows promise for assessing wheelchair propulsion in daily life.

## Introduction

For wheelchair users, maintaining an active lifestyle is generally associated with a decreased risk for cardiovascular diseases, diabetes or pressure ulcers.^1–3^ Simultaneously, over-use of the shoulders and resulting pain or injuries is a paramount concern for many, especially manual wheelchair users who fully rely on their upper extremities for mobility and independence. In fact, the shoulder is the most common site of musculoskeletal pain among manual wheelchair users and, as a result, a plethora of studies have investigated the aetiologies of respective injuries as well as optimal strategies for prevention, intervention, or rehabilitation.^4–9^ This body of research heavily relies on accurate information regarding the forces exerted by the upper extremities on wheelchair push-rims as well as the derived loading conditions that occur in the shoulder joint. To support this effort, wheelchair wheels have been instrumented with force sensors.^10–19^ Most notably, the SMART^Wheel12^ and, later, the Optipush^18^ systems have provided 3D kinetic data in many studies over the last 20 years, but since these are no longer commercially available, access to knowledge on the functional loading conditions in the upper extremities during dynamic activities has been limited to research developments.

A recurring principle in the design of such measurement wheels is separating the push-rim from the wheel-rim and directly mounting it to the hub via an optimally rigid sensor unit. In some cases, off-the-shelf 3D force transducers have been used,^11,15^ while others have applied strain gauges directly to solid metal spokes connecting the push-rim to the wheel hubs.^10,13,16^ More recently, Miyazaki and colleagues used four 2D load cells between a sports push-rim and a disc-shaped adapter plate to allow the measurement unit to be mounted to different sports wheelchair wheels as desired.^17^

A highly stiff connection across the gap between the push-rim and the wheel hub is a common requirement in these different approaches, resulting in substantial bulk and weight in the range of 2.8 kg for the measurement unit^17^ to a total of 5 to 6 kg.^18,20^ The resulting difference in inertia, however, has been shown to influence wheelchair propulsion patterns^20^ and, from a wheelchair user’s perspective, limits the wheels’ usability to applications in confined laboratory environments: heavy wheels are not only exhausting to accelerate and difficult to transfer into cars, but are also obstructive due to the overall increased width of the wheelchair.

If the components required for push-rim force measurement were accurate and lightweight, measurements outside of biomechanical laboratories might become possible, hence unlocking a new thread of research towards understanding shoulder loading patterns during activities of daily living and over longer periods of time. Moreover, such wheels could be used to monitor physical activity or rehabilitation progress outside of clinical settings or for performance diagnostics in para-sports, possibly supporting improvements in training programmes or athlete motivation. The goal of this study was therefore to develop an instrumented wheelchair wheel, focussing on a compact and lightweight design by minimising the need for auxiliary structures.

## Methods

### Design: electronics and sensor layout

**Figure 1.**
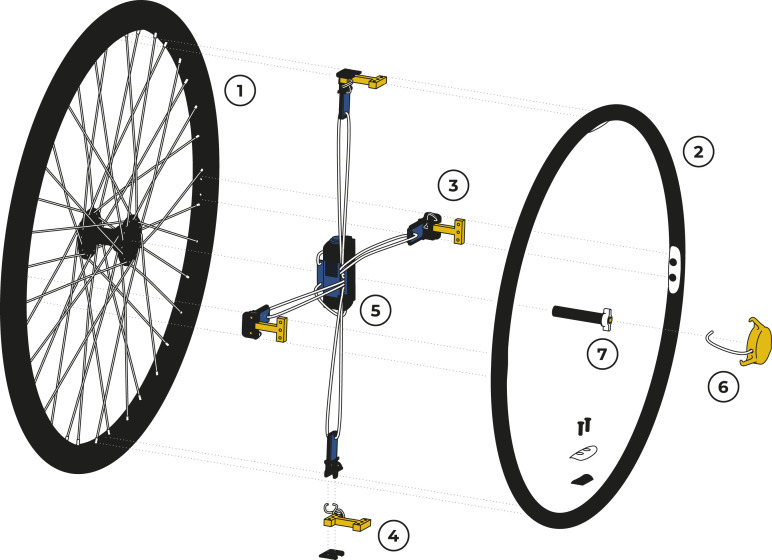
Components of the lightweight instrumented wheelchair wheel: A standard wheelchair wheel (1) and a titanium push-rim (2) were connected directly by sensor units (3) based on custom-made 2D load cells (4). A microcontroller and rechargeable battery were mounted to the central hub (5). Wheel rotation was measured by a rotary Hall sensor (6) placed over the tip of the wheel axle that was equipped with a magnet (7) for reference.

The driving principle to minimise weight in the novel measurement wheel design was to use a standard wheelchair wheel with a titanium push-rim and to instrument four connecting joints. In most previous projects aiming at force measurements on wheelchair push-rims, direct connections between hub and push-rims were equipped with strain gauges, whereas here, custom-made load-cells were developed to attach the push-rim directly to the wheel-rim (Figure 1). These H-shaped aluminium units were dimensioned for a maximum load of 20 kg and acted as both push-rim mounts as well as resistive 2D-load cells. 2 pairs of strain gauges were glued to them, one oriented radially and one tangentially. This arrangement allowed small overall dimensions and the use of lightweight materials for the central sensor units at the cost of omitting axial force measurements (due to limited space availability), resulting in a 2-dimensional measurement instrument with an estimated weight capacity of 80 kg. Resistance changes caused by deformation were amplified using half Wheatstone bridges and conditioned on HX711 analog-to-digital converters (Avia Semiconductor, China) that were mounted directly onto the ends of the load cells, keeping the wire connections as short as possible to avoid unnecessary signal noise. The resulting 8 load signals were processed on a HUZZAH32 ESP32 Feather Board (Adafruit, USA) mounted to the wheel hub on a simple, 3D-printed bracket with a built-in holder for an 18,650 Li-Ion battery (3000mAh). Even though not particularly energy-efficient, use of this open-source system allowed rapid software development and easy wireless communication (Bluetooth Classic) to transmit data at a frequency of 140 Hz to a mobile recording device from two measurement wheels simultaneously.

The wheel was attached to a wheelchair using a split-end axle equipped with a magnet. Once tightened, the axle is unable move or rotate relative to the wheelchair frame and therefore acts a as reference to capture the rotation angle using a Rotary Hall Sensor (55,300 Flat Pack, Littelfuse, USA) placed over the tip of the axle on the hub.

### Signal processing

**Figure 2.**
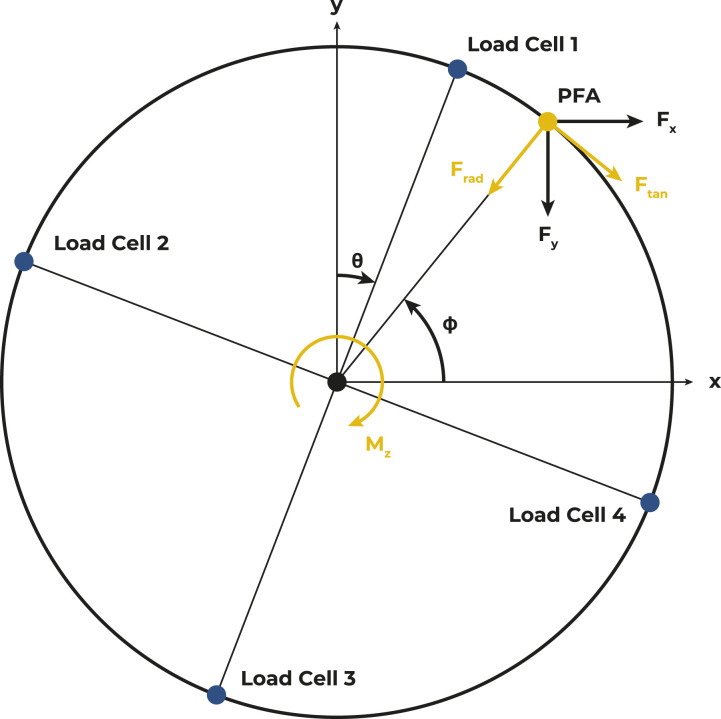
Load cell arrangement and essential parameters calculated from the force signals, where point of force application is the point of force application of the hand force on the push-rim.

Forces and moments applied to the wheelchair push-rim can be illustrated in either an absolute cartesian coordinate system as *F*_*x/y*_ or in a relative polar coordinate system as *F*_*t/r*_ (Figure 2) depending on the research question.^21^ For both expressions, transformations from the load cell signals *V*_ti_ (tangential) and *V*_ri_ (radial) are needed (*i* = (1, 2, 3, 4) representing the load cell number). As a basic convention, load cell 1 was located at the top of the push-rim in the starting position and its angular position was denoted by θ. Before assembly, each load cell was calibrated by hanging 5 kg, 10 kg and 20 kg weights on the cells that were rigidly mounted to a table in order to identify calibration coefficients *k*_ti_ for tangential and *k*_ri_ for radial directions.

Based on the measured strains *V*_ti/ri_ and the position of the load cells θ as well as the radius of the push-rim *R*, the horizontal and vertical force components *F*_*x*_ and *F*_*y*_ as well as the moment around *z* axis *M*_*z*_ can be calculated as follows:

(1) $$\left(\begin{array}{c} F_{x}\\ F_{y}\\ M_{z} \end{array}\right)=\left(\begin{array}{c} \cos\theta \\ \sin\theta \\ R \end{array}\;\begin{array}{c} -\sin\theta \\ \cos\theta \\ 0 \end{array}\;\begin{array}{c} -\sin\theta \\ \cos\theta \\ R \end{array}\;\begin{array}{c} -\cos\theta \\ -\sin\theta \\ 0 \end{array}\;\begin{array}{c} -\cos\theta \\ -\sin\theta \\ R \end{array}\;\begin{array}{c} \sin\theta \\ -\cos\theta \\ 0 \end{array}\;\begin{array}{c} \sin\theta \\ -\cos\theta \\ R \end{array}\;\begin{array}{c} \cos\theta \\ \sin\theta \\ 0 \end{array}\right)\left(\begin{array}{c} k_{t1}V_{t1}\\ k_{r1}\;V_{r1}\\ k_{t2}V_{t2}\\ k_{r2}V_{r2}\\ k_{t3}\;V_{t3}\\ k_{r3}V_{r3}\\ k_{t4}\;V_{t4}\\ k_{r4}V_{r4} \end{array}\right)$$

Before any further calculations, the weight of the push-rim ($m_{p}$) was corrected for:

(2) $${F_{x}}^{\prime }=F_{x}-\left(m_{p}\;g\;\sin\theta \right)$$

(3) $${F_{y}}^{\prime }=F_{y}-\left(m_{p}\;g\;\left(1-\cos\theta \right)\right)$$

Assuming that the torque exerted by the wrist is negligible^22,23^ for calculating *F*_*t*_ and *F*_*r*_, the tangential force component is given by:

(4) $$F_{t}=\frac{M_{z}}{R}$$

The total force *F* on the push-rim for both the absolute and relative system, is given by:

(5) $$F=\sqrt{F_{x}^{2}+F_{y}^{2}}=\sqrt{F_{t}^{2}+F_{r}^{2}}$$

Assuming that the force is always applied in the upper half of the push-rim (ϕ = [0, 180°]), the sign of *F*_*r*_ corresponds to the sign of *F*_*y*_ which allows a unique radial force component to be calculated:

(6) $$F_{r}=\sqrt{F^{2}-F_{t}^{2}}\cdot sign\left(F_{y}\right)$$

With all force components available and, again, assuming the forces are only applied in the upper half of the wheel, the point of force application (PFA), ϕ, can then be estimated:

(7) $$\phi =\cos^{-1}\left(-\frac{F_{r}\;F_{x}-F_{t}\;F_{y}}{F^{2}}\right)$$

As essential parameters, the wheel rotation angle (θ), *F*_*t*_, *F*_*r*_ and the PFA (ϕ) allow the calculation of velocity, exerted power in the forward and backward directions, and the resulting work over time, as well as an estimation of the fraction of effective force or key push characteristics.

### Static validation

**Figure 3.**
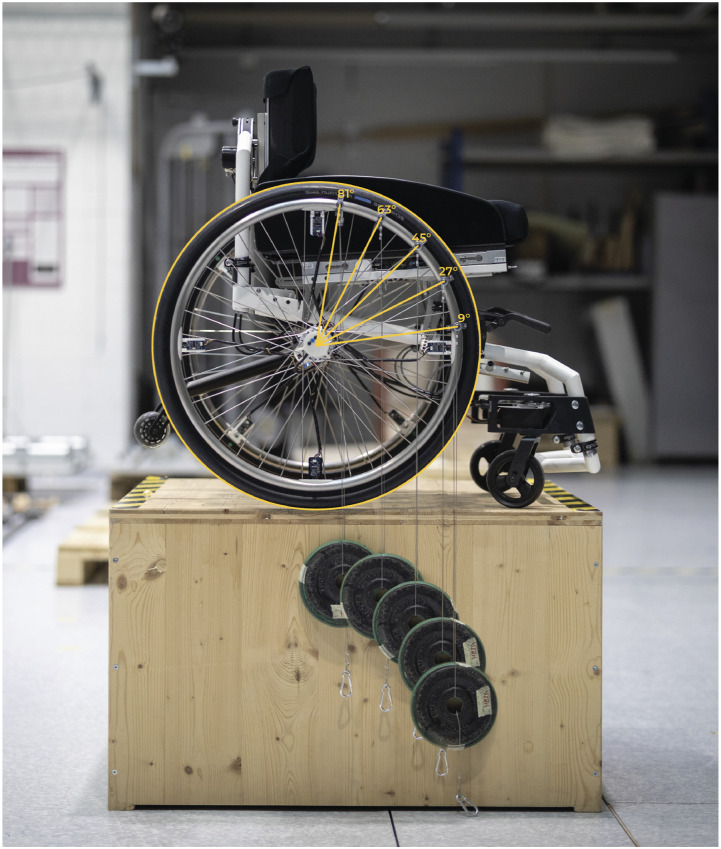
Known weights were hung on the push-rim at known positions for static validation of the lightweight instrumented wheelchair wheel.

For an initial, static validation, the measurement wheel was mounted to a wheelchair and locked at θ = 0°. The wheel was then calibrated by subtracting current sensor offsets from measured values. Weights of mass 2.5, 5 and 10 kg were then hung on the wheel at ϕ = (9°, 27°, 45°, 63°, 81°), three times in each position (Figure 3) to capture the variance caused by different attachment points relative to the sensor positions.

The entire procedure was repeated with the weights attached at a constant position relative to the wheel at θ = 45° (and ϕ = 45°). Instead of varying ϕ by moving the attachment point, the entire wheel was rotated forward and backward in order reach the same PFAs but at different wheel rotation angles θ = (−36°, −18°, 0°, 18°, 36°). This second set of values therefore incorporates rotary sensor signals (and resulting errors) in the calculations. As a given weight should result in the same output value, irrespective of sensor position and wheel rotation angle, the two datasets were consolidated for simplicity.

Theoretical target values were pre-determined for the different mounting angles to allow calculating mean measurement error, error standard deviation as well as a root-mean-square-error for every position and every weight.

## Results

### Wheel implementation

**Figure 4.**
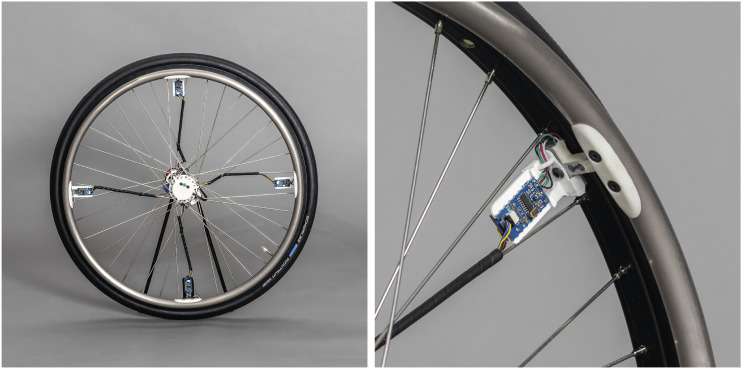
Implementation of the lightweight instrumented wheelchair wheel based on the principle of instrumenting the connector between push-rim.

The sensor arrangement was implemented on a 36-spoke 24″ wheelchair wheel (Küschall, Starec, Invacare International GmbH, Switzerland) using rapid prototyping methods (Figure 4).

In total, the added components for instrumentation weighed approximately 350 g, resulting in a total mass for the wheel of 2.1 kg. For mounting/dismounting it on a wheelchair, the rotary sensor clip is removed from the hub providing access to the 12.7 mm axle with the magnet. The electronic system achieved a battery life of roughly 8 hrs on a single charge.

### Static validation

The calculated force errors were relatively lower at lower loads, whereby the tangential force component showed lower relative errors than the radial force component overall (Table 1). Both tangential and radial force errors – and with them the errors for the PFA – were generally greatest at ϕ = 9°, where the radial force component was smallest, and the tangential force component prevailed. Overall, measurements were most accurate when the applied forces were distributed evenly among the radial and tangential sensors.

**Table 1.** Results from the static validation of tangential and radial force as well as PFA measurements. Target values, mean errors (M E), error standard deviation (E StD) and root-mean-square-error (RMSE) were calculated based on 6 values for each position and load.

		Tangential force [N]				Radial force [N]				Point of force Application [°]			
Weight [kg]	Position [°]	Target	M E	E StD	RMSE	Target	M E	E StD	RMSE	Target	M E	E StD	RMSE
2.5	81	3.9	−0.4	0.3	0.4	24.5	0.3	0.2	0.4	81.0	−1.3	4.4	4.2
2.5	63	11.3	0.0	0.3	0.3	22.1	0.4	0.4	0.5	63.0	−2.6	3.3	4.0
2.5	45	17.6	−0.1	0.3	0.3	17.6	0.3	0.3	0.4	45.0	0.6	2.1	2.0
2.5	27	22.1	0.4	0.2	0.4	11.3	−0.3	0.3	0.4	27.0	3.9	6.8	7.3
2.5	9	24.5	1.4	0.1	1.4	3.9	−4.8	0.2	4.8	9.0	−2.5	11.8	11.1
5	81	7.6	−1.1	0.7	1.2	48.1	0.4	0.2	0.5	81.0	1.0	1.6	1.8
5	63	22.1	−0.5	0.6	0.8	43.4	0.6	0.6	0.8	63.0	−0.5	1.1	1.1
5	45	34.5	0.0	0.4	0.3	34.5	0.2	0.4	0.5	45.0	−1.3	1.5	1.9
5	27	43.4	0.6	0.1	0.6	22.1	−0.8	0.2	0.8	27.0	3.5	7.5	7.7
5	9	48.1	1.2	0.8	1.4	7.6	−4.4	3.9	5.7	9.0	2.9	5.6	5.9
10	81	15.4	−3.1	3.2	4.3	97.3	1.1	1.1	1.5	81.0	0.5	2.9	2.7
10	63	44.7	−1.9	3.2	3.5	87.8	1.5	2.1	2.4	63.0	−1.4	1.0	1.7
10	45	69.6	−0.5	1.2	1.3	69.6	1.1	1.6	1.8	45.0	0.0	3.5	3.2
10	27	87.8	0.4	0.8	0.8	44.7	−0.4	1.5	1.4	27.0	3.8	5.9	6.6
10	9	97.3	3.1	1.3	3.3	15.4	−12.0	5.3	12.9	9.0	0.7	7.2	6.6
Mean			0.0	1.8	1.8		−1.1	3.8	4.0		0.5	5.3	5.3

## Discussion

In recent decades, several research groups have presented instrumented wheels with force sensors to support wheelchair propulsion research, promote healthier physical activities, and prevent shoulder injuries among wheelchair users. Due to the considerable added bulk and weight, measurement wheels have almost exclusively been used in confined laboratory or clinical environments and have been shown to influence propulsion patterns. Taking first steps towards force measurements in daily life to unlock force and activity monitoring in functional settings, the aim of this project was to design, implement and statically validate a prototype lightweight force measurement wheel. The central design principle was to equip the joints between the push-rim and wheel-rim with force sensors, rather than a designated connection between push-rim and wheel hub. The components needed for instrumentation added only approximately 350 g to a standard wheelchair wheel keeping the overall weight at a fraction of previous solutions: with 2.1 kg total mass, the prototype is more than 50% lighter than a SMART^Wheel^,^20^ although it should be acknowledged that some of the weight reduction of the novel design has been achieved by omitting axial force measurements that were reported to only account for up to 10% of the resultant force.^24^ Still, instrumenting the joints between the push-rim and wheel-rim appears to be a promising principle for future developments that should aim at improved accuracy and better integration of electronic components to enable tracking wheelchair users’ activities in real life settings.

The omission of axial forces not only reduces the system’s complexity, but certainly also limits its applicability in advanced research projects. Especially when investigating loading conditions in the shoulder joints, for instance through musculoskeletal modelling, researchers rely on accurate 3D-data. However, for many endeavours where power output, overall work, push angles, or push times are of interest, 2-dimensional – in fact, in many cases even 1-dimensional (tangential) – measurements are sufficient.^25^

The reduction in dimensionality further necessitated some assumptions for the calculation of essential force components that have affected the measurement accuracy. With mean errors of up to ±3.1 N for forces in the range and positions of propulsive pushes, tangential and radial forces were still comparable to values reported by Kim et al. for their bilateral measurement system,^14^ or those measured by Guo and colleagues for the OptiPush wheel.^18^ Measurement errors were lower under high loads, likely due to the lower, percental amount of signal noise. Highest errors were found when forces were applied at the frontal extreme of the wheel where the hung weight almost exclusively exerted tangential forces onto the push-rim, but such conditions are unlikely to occur during actual wheeling. Similarly, the PFA estimation showed considerable errors up to more than 10°. While this is still comparable to errors originally reported for the SMART^Wheel^,^10^ it is justifiable to expect better accuracy from up-to-date measurement technologies. As the PFA is determined based on previously calculated forces, it is, of course, subject to error propagation. Inclusion of the 3^rd^ measurement dimension to the sensor units would therefore appear to not only provide more comprehensive datasets, but also improve overall accuracy by eliminating the need for assumptions, as well as allow wrist moments to be included in the calculations. Unsurprisingly there seems to be a trade-off between accuracy and spatial resolution versus the bulkiness of the system. Future projects should therefore be clear about their requirements and develop application-specific instruments.

Finally, previous authors have presented static measurements as a baseline validation for their measurement instruments.^15,17,19^ While methodically comparable in that respect, the lack of dynamic validation measurements is a clear limitation of this project at this stage. Towards improving validity, future developments should identify methods that are able to mimic the dynamic, cyclic characteristics of wheelchair propulsion in a controlled and repeatable manner for validation and to ultimately pave the way for functional measurements in real life settings.

## Conclusion

In this project, a lightweight instrumented wheelchair wheel for force measurements was developed and statically validated. The principle of instrumenting the joints between push-rim and wheel-rim shows promise for considerable reduction in the overall weight and bulk of measurement wheels. The reduction to a 2-dimensional measurement system further simplified the overall design but entailed a series of assumptions for the calculation of essential parameters that affect measurement accuracy. Future developments should focus on application-specific instruments to either improve measurement accuracy or aim at better integration of components to further expand the understanding of physical activities in wheelchair users’ lives.
